# Addressing the challenge of tuberculosis screening in diabetic populations: a narrative review of methods and strategies

**DOI:** 10.3389/fpubh.2025.1703990

**Published:** 2025-12-02

**Authors:** Yuqi Ma, Xiwei Lu, Xu Cao, Xintong Lv, Jiaying Li

**Affiliations:** 1 Department of Public Health, Dalian Medical University, Liaoning, China; 2 Research Institute for Public Health, Dalian Public Health Clinical Center, Liaoning, China

**Keywords:** tuberculosis, diabetes, co-morbidity of diabetes and tuberculosis, screening methods, effectiveness evaluation

## Abstract

The comorbidity of diabetes and tuberculosis (TB) poses a major public health challenge. Patients with diabetes have impaired immune function, increasing their risk of contracting TB. They often present with atypical clinical symptoms after infection, which can lead to delayed diagnosis. Therefore, TB screening in the diabetic patients is essential. This study systematically reviews advances in TB screening strategies for diabetic patients by searching databases including PubMed, WHO Global Index Medicus, Web of Science, China National Knowledge Infrastructure (CNKI), and Wanfang Data Knowledge Service Platform (search period: 2000–2025). We compare the efficacy of various screening techniques and strategies, discuss their suitable scenarios, advantages, and disadvantages, and provide recommendations for post-screening case management and cohort studies of screened populations. Our review found that novel screening technologies are gaining traction for TB screening in diabetes. AI-based imaging significantly improves the accuracy and efficiency of traditional radiological diagnosis. Oral swab testing and urine-based lipoarabinomannan (LAM) testing help overcome the challenge of specimen collection in individuals who have difficulty producing sputum. Recombinant fusion protein ESAT6-CPF10 (EC) skin test shows good accuracy and cost-effectiveness for latent tuberculosis infection (LTBI) screening. However, some techniques still require large-scale validation specifically in diabetic patients. The choice of TB screening strategy should consider local TB prevalence, prioritizing screening for high-risk subgroups among diabetic patients is cost-effective. The therapeutic management of patients with concurrent diabetes and tuberculosis is challenging. Successful treatment depends on the appropriate management of drug interactions, improved medication adherence, effective glycemic control, and timely drug susceptibility testing to adjust anti-TB regimens. Preventive treatment regimens for patients with diabetes and LTBI require further research. This study aims to provide evidence-based references for public health policymakers to help develop more targeted TB screening systems for diabetic patients, thereby improving early diagnosis and treatment rates, and reducing TB incidence and transmission risk.

## 1 Introduction

Tuberculosis (TB) is a chronic infectious disease caused by *Mycobacterium tuberculosis* (Mtb). The World Health Organization (WHO)'s 2024 Global Tuberculosis Report indicates approximately 10.8 million new TB cases globally in 2023, with an incidence rate of 134 per 100,000 population, a 4.6% increase compared to 2020 (1). Diabetes mellitus (DM) is a group of chronic metabolic disorders characterized by persistent hyperglycemia, arising from multiple etiologies. TB and diabetes are closely linked and influence each other. Diabetes increases the risk of TB treatment failure and drug resistance, while the oxidative stress associated with TB worsens insulin resistance (2). This relationship increases the overall disease burden and poses a significant obstacle to achieving the global goal of “Ending TB by 2035” (3). Therefore, TB screening in diabetic patients has become a core strategy for the early detection of cases and interrupting transmission chains, and it has been recommended by the WHO. However, TB screening in diabetic patients faces several technical challenges. It is necessary to evaluate the accuracy, portability, and cost-effectiveness of different screening techniques through comparative analyses in order to select and develop optimal strategies. Although existing guidelines emphasize the necessity of TB screening in diabetic patients, they fail to provide differentiated screening pathways that account for regional healthcare resources and diabetic population stratification. Consequently, this review aims to synthesize the research evidence on TB screening among diabetic patients, with a focus on integrating the performance of traditional and novel screening technologies and practical experiences from different implementation settings. It also explores risk stratification-based screening strategies and post-screening management. The goal is to provide more targeted, evidence-based references for public health decision-making.

## 2 Methods

This narrative review was conducted through a systematic search of the PubMed, WHO Global Index Medicus, Web of Science, China National Knowledge Infrastructure (CNKI), and Wanfang Data Knowledge Service Platform databases, covering the period from 2000 to 2025. The search strategy combined keywords and their abbreviations, including core terms such as “diabetes (mellitus),” “tuberculosis,” “latent tuberculosis infection,” “screening,” “DM,” “TB,” and “LTBI.” The search was not restricted by region or language. We included randomized controlled trials, cohort studies, cross-sectional studies, systematic reviews/meta-analyses, and guidelines from authoritative institutions like the World Health Organization (WHO). The selected studies focused on people with diabetes and investigated tuberculosis or latent tuberculosis infection screening methods, effectiveness, risk stratification, and the application of artificial intelligence in screening and diagnosis. After the screening process, 109 articles in Chinese and English were included.

## 3 Epidemiological association between diabetes and tuberculosis

According to the International Diabetes Federation (IDF) 2024 report, approximately 589 million adults worldwide have diabetes, a number projected to reach 853 million by 2050 (4). Compared to people without diabetes, those with diabetes have a 2- to 4-fold higher risk of developing active tuberculosis and a 1.18-fold higher risk of latent tuberculosis infection (LTBI) progression (5, 6). Most cases of DM-TB comorbidity are found in high TB burden countries such as India, China, and Nigeria (7). A dynamic mathematical model of tuberculosis and diabetes in Indonesia projected that the proportion of TB cases attributable to diabetes would increase from 18.8% in 2020 to 20.9% in 2030 and 25.8% in 2050. Similarly, the proportion of TB-related deaths attributable to diabetes was projected to rise from 24.3% in 2020 to 27.7% in 2030 and 34.3% in 2050. Over the next 30 years, diabetes is expected to become a major driver of TB incidence and mortality (8). A meta-analysis reported a 7.2% prevalence of diabetes and pulmonary tuberculosis (PTB) co-infection in China, with notable epidemiological disparities: higher rates in northern regions than southern regions (9.13% vs. 5.94%), in individuals aged ≥40 years than in those <40 years (12.18% vs. 2.33%), and among smear-positive PTB cases than smear-negative cases (11.4% vs. 4.0%) (9). The bidirectional relationship between diabetes and tuberculosis complicates their epidemiological association. On one hand, diabetes increases susceptibility to tuberculosis by impairing immune functions, such as cell-mediated and humoral immunity (6, 10, 11). Diabetic patients are also more likely to develop drug-resistant tuberculosis, which increases treatment failure rates and mortality (12). On the other hand, tuberculosis can exacerbate metabolic disorders in diabetes, leading to further elevation of blood glucose levels after infection and thereby forming a vicious cycle (2).

## 4 Target population for tuberculosis screening among diabetic patients

The World Health Organization (WHO) recommends TB screening for diabetic patients in its 2021 “WHO Consolidated Guidelines on Tuberculosis: Module 2: Screening—Systematic Screening for Tuberculosis Disease” (13). TB screening should be prioritized in high-burden areas (incidence ≥100 per 100,000) (14). In low-burden areas or resource-limited regions with high TB prevalence, the cost-effectiveness of screening strategies should be carefully evaluated (15). A three-year evaluation of community-based active TB screening in Vietnam showed that, in TB-endemic areas, annual community-wide screening was more effective than passive case-finding through symptomatic presentations in reducing TB prevalence. The prevalence of PTB decreased from 389 per 100,000 in the first year to 126 per 100,000 by the fourth year (16). In contrast, a population-based cohort study in the UK found a TB prevalence of only 87.28 per 100,000 among diabetic patients (17), and therefore routine TB screening in this population is not recommended. Consequently, TB screening strategies for people with diabetes should be tailored to regional epidemiology and cost-effectiveness. Initiating screening and health management for high-risk groups in resource-limited, moderate to high TB prevalence regions is likely to be a cost-effective approach (15).

The effectiveness of TB screening among diabetic patients varies across China due to significant differences in TB prevalence between provinces. A 2013 community-based TB screening study of individuals aged 25 and above with type 2 diabetes in Shandong Province reported a TB detection rate of 342.7 per 100,000 (18). In contrast, TB screening in a low-prevalence area, such as Shanghai’s Xuhui District in 2015, was less efficient, with a detection rate of only 31.39 per 100,000 among diabetic patients aged 15 and older (19). Stratifying the risk of developing active TB among diabetic patients is highly valuable. Studies have identified several high-risk factors, including male sex, age ≥65 years, low BMI (<18.5 kg/m^2^), smoking, HbA1c > 7% or fasting plasma glucose (FPG) ≥ 7 mmol/L, and a diabetes duration of more than 10 years (20–25). The risk is further increased by a history of TB contact, HIV co-infection, or cirrhosis (23, 26). People with diabetes in rural areas show higher TB detection rates, which is attributed to lower health awareness and insufficient healthcare access (27). Screening strategies should be tailored to local epidemiology. A TB screening study in Sri Lanka found that diabetic males with uncontrolled diabetes (HbA1c > 8%) who are over 60 years of age had a TB detection rate seven times higher than the general population (28), supporting targeted screening for this group. Research from Jiangsu Province, China, suggests that screening people with diabetes who have specific symptoms (such as cough, sputum production, or weight loss) and an FPG ≥ 12 mmol/L is more cost-effective (29). In summary, cohort studies to identify local risk factors for TB among diabetic patients can inform the development of targeted screening strategies and improve efficiency. Machine learning-based tools for the dynamic risk assessment of follow-up health data in regional diabetic patients promise to be valuable for formulating future screening strategies.

LTBI is a major source of active TB cases. The World Health Organization (WHO) 2023 Consolidated Guidelines on Tuberculosis, Module 1: Prevention—Tuberculosis Preventive Treatment, explicitly states that screening for LTBI is not recommended for diabetic patients alone, unless they are members of high-risk groups such as HIV-infected individuals or household close contacts (30). However, recent multicenter studies have confirmed that people with diabetes, particularly those with poor glycemic control or longer disease duration, have a significantly higher risk of LTBI progressing to active TB than the general population (31). The 2024 updated guidelines revised this recommendation by removing the previous restriction on LTBI screening in people with diabetes, although no specific new recommendation was provided. To date, there is a lack of high-quality evidence supporting the effectiveness of LTBI screening and preventive treatment in reducing TB incidence among diabetic patients. Some researchers suggest that in high TB burden regions, LTBI screening should be implemented alongside active TB screening in high-risk diabetic subgroups. They also recommend conducting research on the protective effect of preventive treatment in these individuals with LTBI, which would help develop a comprehensive strategy integrating TB screening, treatment, and management in this population (32, 33).

## 5 Evaluation of tuberculosis screening methods in the diabetic patients

Tuberculosis screening methods include symptom screening, imaging screening, pathogen screening, and immunological screening. Typically, a combination of screening methods is required to complete the screening process. With technological advancements, molecular biology detection techniques and artificial intelligence (AI)-based imaging detection can improve screening efficiency, significantly reduce screening costs, and hold broad prospects for on-site screening. The comparison of screening techniques is shown in Table 1.

**Table 1:** Comparison of screening technologies.

Technology category	Detection technology	Advantage	Disadvantage	Applicable scenarios	Cost-effectiveness
Imaging examination	CXR	Simple operation; affordable	Limited sensitivity; difficult to detect early, mild lesions and bronchial mucosal tuberculosis	Large-scale initial screening of diabetic populations	Low cost, suitable for resource-limited areas, but has a high rate of missed diagnoses when used alone; requires integration with other methods to enhance effectiveness
	Chest CT	High resolution, strong ability to detect occult lesions	Conventional CT scans involve high radiation exposure and high costs, making them unsuitable for large-scale screening	Auxiliary diagnosis of abnormal shadows on CXR	High cost, suitable only as an auxiliary diagnostic tool, and not cost-effective for large-scale screening
	AI + CXR	Improve diagnostic accuracy and efficiency; reduce missed diagnoses caused by human fatigue	Insufficient specificity, relatively high false positive rate	Tuberculosis-endemic regions with limited radiology resources	Reduce labor costs, but the algorithm needs to be optimized to improve specificity, with high initial R&D costs
Pathogen detection	Sputum smear	Simple and quick operation; low cost	Low sensitivity, unable to distinguish NTM infection	Further evaluation of abnormal shadows on CXR	Low cost, but high rates of missed diagnoses lead to increased subsequent treatment expenses
	Sputum culture	High specificity and sensitivity; the gold standard for tuberculosis diagnosis	Long testing cycles and high costs; high demands on laboratory environment and technical expertise, prone to contamination	The definitive basis for diagnosing tuberculosis	High time cost, not suitable for on-site screening
	GeneXpert MTB/RIF	Rapid Detection (1–2 h); simultaneous detection of rifampicin resistance; high sensitivity and specificity	The testing fees are relatively high	Suitable for primary healthcare facilities; diagnosis and drug resistance testing for suspected TB patients	Although the cost per test is high, rapid diagnosis can reduce transmission, making it cost-effective for diabetic populations and resource-limited areas
	TB-LAMP	No thermal cycler required, amplification at constant temperature simplifies operation; sensitivity and specificity exceed sputum smears, comparable to GeneXpert	Susceptible to aerosol contamination leading to false positives; requires high-standard laboratory environments, involves high reagent costs, and necessitates operation and interpretation by trained professionals	As an alternative to sputum smears in settings lacking GeneXpert equipment (It has not been validated in diabetic populations)	The cost is higher than sputum smear testing, but the overall cost-effectiveness is comparable to GeneXpert
	Oral swab testing	Non-invasive, quick, and simple operation; suitable for those with difficulty producing sputum	Sensitivity is lower than that of sputum samples; false positives may occur due to contamination or improper collection	Suitable for various settings, including communities, clinics, and large medical facilities	Low operating costs, but poor sensitivity, requires combination with other methods, cost-effectiveness is generally average when used alone
Immunological testing	TST	Low cost; simple operation	Affected by BCG vaccination and most NTM infections; false negatives are common in diabetic populations; not to be used alone for TB screening	Screening for LTBI	Low cost, but accuracy is interrupted. It has limited effectiveness in diabetic populations, it cannot serve as an independent tool for TB case screening
	IGRA	High accuracy; unaffected by BCG vaccination and most NTM infections	High cost, requires laboratory testing; unable to distinguish between LTBI and ATB; not to be used alone for TB diagnosis	Screening for LTBI	Higher cost, exhibits advantages over TST in LTBI screening, but demonstrates poor cost-effectiveness in TB case detection
	ECST	Low cost and simple operation; higher sensitivity and specificity than TST; unaffected by BCG vaccination and most NTM infections	Individuals with impaired immune systems may produce false-negative results	Screening for LTBI (Further clinical validation is required)	Low cost, high potential, but currently lacking large-scale cost data
	Urine-based LAM	Simple operation, low cost, non-invasive, suitable those with difficulty producing sputum	Low sensitivity; not used as a routine screening tool domestically, requires technical optimization	Initial screening for diabetic patients in primary healthcare settings	Low cost, offering cost-effectiveness in specific populations (such as for HIV-infected individuals), but requires combination with other methods to improve sensitivity

NTM, Non-Tuberculous Mycobacteria.

### 5.1 Imaging examination

Chest X-ray (CXR) is a standard screening tool for PTB due to its low cost. Mobile digital radiography (DR) units are the primary tool for on-site screening. Patients with suspected TB on CXR are referred for chest computed tomography (CT) or microbiological tests for further diagnosis. The main limitation of CXR screening is its difficulty in detecting early or mild lesions, lesions in concealed locations, and bronchial tuberculosis. Chest CT, with its high resolution, provides detailed lung images and significantly improves the detection of TB lesions, especially those in concealed locations. However, due to higher radiation exposure and cost, conventional CT is not suitable for large-scale screening (34). It is best used as a supplementary imaging tool following abnormal CXR findings. Concurrent TB screening during low-dose computed tomography (LDCT) screening for lung cancer has also been proposed as a valuable approach (35).

Artificial intelligence (AI) is advancing rapidly in medicine and shows great promise for TB screening. AI-assisted imaging, such as computer-aided detection (CAD) software, can generate real-time diagnostic reports, enabling remote interpretation by radiologists. This approach reduces the demand for extensive healthcare resources and improves the accuracy and efficiency of TB diagnosis, proving particularly valuable in high-burden areas with a shortage of radiologists. A study from Pakistan evaluating CAD4TB demonstrated good diagnostic accuracy as a triage tool for TB screening among diabetic patients (36). A systematic review on CAD showed its high potential for active TB screening in diabetic patients, reporting a pooled sensitivity of 0.94 (95% confidence interval [CI]: 0.85–0.97) and a pooled specificity of 0.77 (95% CI: 0.68–0.84) across four different software algorithms (37). Despite these advantages, AI systems still face challenges of high sensitivity but insufficient specificity. Follow-up and investigation of screen-positive patients still require substantial effort, and currently, no CAD product meets the WHO’s minimum ideal diagnostic performance criteria for TB screening (38).

### 5.2 Pathogen detection

Pathogen detection is the primary basis for a definitive TB diagnosis. Sputum smear microscopy has low sensitivity, with a smear-positive detection rate of only 30–50% (39). Using AI-based Ziehl-Neelsen (ZN) or Auramine-O/Auramine Rhodamine fluorescent staining to automatically detect acid-fast bacilli in sputum samples can improve testing throughput and sensitivity (40). However, traditional pathogen detection is difficult to implement at scale in mass screening programs. Both smear and culture for Mtb require biosafety-level laboratories, making them challenging to deploy rapidly in on-site screening. Generally, sputum smear and culture are routinely performed for suspected cases identified through CXR screening. Sputum culture is the “gold standard” for TB diagnosis, with high specificity and sensitivity, but it takes 4–8 weeks to obtain results, failing to meet the demand for rapid clinical diagnosis and screening (41).

Nucleic acid amplification tests (NAAT) can rapidly detect Mtb DNA using various polymerase chain reaction (PCR) amplification methods. Among them, GeneXpert MTB/RIF is a real-time quantitative PCR assay that simultaneously identifies Mtb and rifampicin resistance. It does not require strict biosafety conditions and can be performed in community health service centers, making it a WHO-recommended diagnostic tool for PTB (42). Thus, even with infrastructural and implementation challenges in low- and middle-income countries, GeneXpert remains the point-of-care (POC) platform for TB diagnosis in healthcare settings (43). A prospective cohort study in India showed that GeneXpert had high sensitivity (96%) and specificity (91%), with excellent diagnostic performance in people with both DM and TB. The sensitivity of GeneXpert was higher in the diabetes group (98%) than in the non-diabetes group (95%), supporting the rapid rollout of this technology in regions with a high dual burden of TB and DM (44).

Loop-mediated isothermal amplification (LAMP) is a nucleic acid amplification technique performed under constant temperature conditions. Compared to smear microscopy, it offers more straightforward operation with higher sensitivity and specificity. WHO guidelines recommend it as an alternative to smear microscopy for diagnosing PTB in adults (45). As it requires no thermal cyclers or fluorescence detection systems, TB-LAMP can be used in primary healthcare or community screening settings, shortening the time to diagnosis (46). A meta-analysis of TB-LAMP diagnostic accuracy reported moderate sensitivity (77.7%) and high specificity (98.1%), with pooled sensitivity and specificity comparable to those of GeneXpert MTB/RIF. In resource-limited settings with insufficient GeneXpert infrastructure, TB-LAMP represents a feasible alternative to smear microscopy (45). The pathogen positivity rate is higher in people with DM and PTB than in those with PTB alone (58.74% vs. 32.52%) (9). Therefore, portable Mtb DNA detection technologies like TB-LAMP hold unique value in TB screening among diabetic patients.

For individuals who have difficulty producing sputum, oral swab testing offers a novel alternative for sample collection. This method uses a swab to collect oral mucosal secretions, which are then tested for Mtb using molecular diagnostics. It is simple, rapid, non-invasive, and suitable for various settings, including communities, clinics, and large medical facilities. A meta-analysis reported a sensitivity range of 36–91% for oral swabs in diagnosing PTB in adults (47). A Ugandan study evaluated a strategy combining tongue swabs with GeneXpert MTB/RIF Ultra in adults with suspected TB. Compared to the reference standard (sputum tested with GeneXpert MTB/RIF Ultra), the dual-swab method using Cepheid sample reagent and Xpert MTB/RIF Ultra processing showed an overall sensitivity of 77.8% (95% CI: 64.4–88) and specificity of 100% (95% CI: 97.2–100) (48). An early Health Technology Assessment (HTA) model from Thailand further indicated that tongue swabs combined with real-time quantitative PCR were more cost-effective than TB-LAMP, particularly in primary healthcare settings (49). In the future, combining this method with AI-based imaging could make it a valuable tool for community-wide screening (50). The primary limitation of oral swabs is their lower sensitivity compared to sputum samples. A clinical study in Uganda found a significantly lower positivity rate for tongue swabs (44%) than for sputum testing (93%) (51). Additionally, false positives may occur due to sample contamination or improper collection (52). Most current studies focus on adults with suspected TB symptoms, with limited research targeting diabetic patients, necessitating further evaluation (53). Nevertheless, given the high pathogen positivity rate in people with DM and TB, oral swabs show promise as a suitable screening technique for this group.

### 5.3 Immunological testing

Immunological tests can help determine tuberculosis infection status. The tuberculin skin test (TST) is based on a type IV hypersensitivity reaction, involving intradermal injection of a small amount of tuberculin purified protein derivative (PPD) to detect prior infection with Mtb. Although TST results can be influenced by BCG vaccination and non-tuberculous mycobacterial infection, its low cost and simple procedure have led to its widespread use in LTBI screening (54). However, it is prone to false-negative results in immunocompromised patients, such as those with diabetes, which reduces its diagnostic value (55). The interferon-gamma release assay (IGRA) is a technique based on Mtb-specific T-cell immunity. It detects interferon-gamma produced by Mtb-specific T cells to determine infection (56). Since it is unaffected by BCG vaccination, IGRA generally has higher accuracy than TST (57, 58). Considering China’s widespread BCG vaccination and cost-effectiveness principles, a two-step strategy—confirming TST-positive results with IGRA—can improve detection accuracy and is highly cost-effective (59, 60). Several countries have incorporated this approach in their national TB control guidelines (61, 62). Nevertheless, neither test reliably distinguishes LTBI from active TB, and both have limited ability to predict disease progression. A meta-analysis showed that in low-TB-incidence countries, IGRA had a stronger predictive ability for progression from LTBI to active TB than TST, with a hazard ratio (HR) of 10.38 (95% CI: 4.17–25.87) for IGRA and 5.36 (95% CI: 3.82–7.51) for TST (5–15 mm induration). But both tests approached the WHO sensitivity target of ≥75%. In high-TB-incidence countries, predictive performance declined, with an HR of 1.61 (95% CI: 1.23–2.10) for IGRA and 1.72 (95% CI: 0.98–3.01) for TST (5–15 mm), making them comparable (63). Overall, neither IGRA nor TST can serve as primary screening tools for case identification.

The recombinant *Mycobacterium tuberculosis* fusion protein skin test (ECST) uses genetically engineered ESAT-6 and CFP-10 fusion proteins to induce a specific delayed-type hypersensitivity reaction, identifying Mtb infection (64). Since these proteins are absent in BCG vaccine strains, ECST minimizes cross-reactivity from BCG vaccination or non-tuberculous mycobacterial infection. The WHO’s 2022 rapid assessment of novel Mtb antigen skin tests (TBSTs) showed that TBSTs, including ECST, had higher accuracy and cost-effectiveness than TST and IGRA, with a safety profile similar to TST (65). A study evaluating ECST’s safety and diagnostic performance further confirmed that ECST demonstrated higher sensitivity and specificity than TST (sensitivity: 89.41% vs. 85.88%; specificity: 96.98% vs. 61.06%). Agreement between ECST and T-SPOT. TB was higher (*κ* = 0.85) than between TST and T-SPOT. TB (κ = 0.28), with an overall favorable safety profile (66). ECST is less costly and more cost-effective for LTBI screening (67). Currently, there is no evaluation of ECST for LTBI screening in diabetic patients, and its ability to predict TB progression remains unclear. Further evidence is needed to support its widespread use in large populations (68, 69).

### 5.4 Urine-based lipoarabinomannan

The lipoarabinomannan (LAM) detection kit is the only rapid test recommended by the WHO for detecting active TB in urine samples (70). Urine-based LAM testing detects LAM antigens released during the breakdown of *Mycobacterium tuberculosis* cell walls. It utilizes methods such as immunochromatography or enzyme-linked immunosorbent assay (ELISA), where antibodies bind to LAM antigens to generate a detectable signal, thereby determining the presence of active TB (70). Urine-based LAM testing overcomes limitations in testing patients unable to produce sputum, offering low cost and simple operation, though with relatively low sensitivity. A domestic study on the diagnostic efficacy of urine-based LAM reported a sensitivity of 46.2% and a specificity of 96.5%. When combined with traditional pathogen detection methods like GeneXpert, it effectively improves detection rates (71). Although currently available urine-based LAM detection methods exhibit overall insufficient sensitivity, they demonstrate higher sensitivity in patients coinfected with HIV. The WHO recommends their use for rapid initial screening in HIV-infected populations (72). This test is suitable for implementation in primary healthcare settings, but it is not recommended for standalone screening. It can be combined with GeneXpert testing to improve sensitivity. Another study evaluating LAM demonstrated that diabetes is an independent risk factor affecting LAM diagnostic sensitivity. Tuberculosis patients with diabetes had approximately three times higher LAM positivity rates compared to non-diabetic tuberculosis patients (OR: 3.14, 95% CI: 1.06–9.29) (73). LAM demonstrates greater specificity for identifying tuberculosis infection in diabetic patients, making it suitable as an initial screening tool for this group. In extrapulmonary tuberculosis cases, LAM detection rates also significantly exceed those of GeneXpert, TB-DNA, and sputum culture (73). Currently, it is not yet adopted as a routine screening tool in China and requires further technical optimization (74).

### 5.5 Combined use of screening schemes

Current TB screening strategies prioritize the integration of multiple diagnostic modalities. For TB screening among diabetic patients, the primary approach involves initial symptom inquiry and CXR detection. Individuals with abnormal CXR findings or positive symptoms undergo sputum testing or rapid molecular testing. This combined screening strategy has been widely implemented in high-burden regions such as China, Nigeria, India, Pakistan, and South Africa. Studies report detection rates ranging from 0.5 to 2.7%, indicating its feasibility. However, most studies lack cost reporting or cost-effectiveness analysis (15, 75–79). A large-scale TB screening program targeting diabetic patients was implemented in Jiangsu Province, China, utilizing CXR as the primary screening tool. Research indicates the feasibility of large-scale TB screening in diabetic patients, but the average cost per TB case detected was $560, resulting in low overall benefits and poor cost-effectiveness (29). In addition, suspected TB patients identified through initial screening require further examinations and must be referred to designated TB medical institutions for confirmation diagnosis. However, poor compliance is commonly observed among these patients, often due to difficulties in accessing medical care or the absence of obvious symptoms. This issue is particularly prominent in remote areas or regions with limited healthcare resources, where the rate of patient attendance at designated institutions is insufficient. Consequently, this leads to underdiagnosis and missed cases of TB. Aside from this typical screening strategy, an Indonesian study used a strategy in which all diabetic patients had CXR. Positive results were subjected to further sputum smear/culture testing based on a CAD4TB risk score of ≥65. This approach achieved more precise patient detection, substantially reduced the proportion of patients requiring laboratory testing, and additional cost savings (80). In a Nigerian active case-finding study among high-risk groups, researchers employed a “mobile computer-aided digital CXR (CAD) screening + GeneXpert molecular confirmation” strategy. This approach increased bacteriological confirmation rates eightfold, from 0.2 to 1.7%, compared to the calibration phase. The number of individuals screened per confirmed case decreased from 8.2 to 7.6, and treatment success rates improved from 70.6 to 83.2%. This mobile screening model effectively increases TB detection rates and improves clinical outcomes, which is particularly suitable for high-burden regions with limited resources, and can serve as a valuable reference for high-burden TB regions worldwide (81).

## 6 Scenario classification for tuberculosis screening among diabetic populations

### 6.1 Community screening

Research indicates that the majority of global TB transmission likely originates from asymptomatic or subclinical disease carriers (82). Community-based TB screening can extend coverage to impoverished, remote, and resource-limited regions, providing opportunities for early diagnosis and treatment while mitigating intra-community TB transmission. The advantage of implementing TB screening among diabetic patients lies in its deep integration with diabetes management frameworks. China has incorporated diabetes management into its basic public health services system. Community health service centers can establish standardized health records to provide organizational, managerial, and follow-up support for large-scale TB screening. This community-based management model not only enhances screening efficiency but also facilitates continuous health management through dynamic updates to health records. Individuals diagnosed with TB during screening can be directly referred to designated medical institutions and enrolled in long-term follow-up programs. Nevertheless, Community-based screening faces several challenges, including limited medical resources, shortages of specialized personnel and rapid diagnostic tools, as well as potential stigma or discrimination at the population level (83). Therefore, optimizing the implementation of community-based TB screening is imperative. Specific optimization strategies include the following:

1. Strengthen health education to dispel TB-related stigma and improve patient participation rates.
2. Implement risk-stratified screening protocols, prioritizing high-risk diabetic patients such as the older adult(s) and those with low BMI (29).
3. Select appropriate screening tools and adopt advanced technologies, such as AI-based imaging and rapid nucleic acid amplification assays, to enhance diagnostic accuracy.
4. Establish an information-sharing platform. Integrating TB registration systems with electronic diabetes health records by adding a “TB-suspicious symptoms” field to health records enables real-time data sharing (84).

However, ongoing evidence regarding the effectiveness of community-based TB screening remains limited, including the long-term impacts of screening cessation and the cumulative benefits of repeated screening rounds (85), necessitating further research.

### 6.2 Diabetes outpatient screening

TB screening in diabetes outpatient settings involves healthcare providers conducting TB screening and differential diagnosis for diabetic patients visiting clinics. It is suitable for centralized patient management and provides an effective environment for early TB detection. A study conducted at a diabetes clinic in the Republic of the Marshall Islands—a small island nation with high rates of both TB and DM—revealed that the incidence of TB over a two-year period was more than 20-fold higher than the general population TB incidence reported in 2012 (86). Diabetes outpatient screening offers high efficiency, access to relatively abundant medical resources, and clinicians with extensive clinical experience, capable of accurately assessing patient conditions, and thereby addressing diagnostic delays. Outpatient settings enable immediate intervention. For diabetic patients presenting for care, clinicians can conduct TB symptom interviews and risk assessments, arrange on-site CXR and sputum molecular testing as clinically indicated, initiate anti-TB treatment for positive cases immediately, and simultaneously adjust diabetes management regimens, thus achieving a closed-loop “diagnosis-treatment-management” system for coexisting TB and DM (87). However, some diabetic patients avoid outpatient visits or refuse TB screening due to low awareness of TB or financial barriers among low-income populations (88). Therefore, policy support from health administration authorities and medical insurance agencies is urgently required to address these barriers. Integrating TB prevention knowledge into routine health education for diabetic patients can enhance their awareness and proactive participation in screening. Meanwhile, medical insurance agencies can revise policies to include TB screening programs within the scope of medical insurance reimbursement, thereby alleviating the financial burden on patients.

### 6.3 Inpatient screening

Inpatients can be centrally managed, ensuring high accessibility to medical resources and improved patient compliance, which facilitates the organization of screening initiatives. This approach enhances the efficiency of bidirectional screening for DM and TB (89). Screening for diabetes among hospitalized TB patients enables the early identification and treatment of individuals with coexisting diabetes, thereby improving TB treatment outcomes and reducing the risk of TB recurrence (90). Conversely, TB screening among hospitalized diabetic patients can leverage electronic health records to automatically identify high-risk individuals, enabling targeted stratified screening and mitigating intra-facility TB transmission. The effectiveness of inpatient screening is closely linked to the establishment of standardized procedures. In the absence of formalized bidirectional screening and implementing relevant protocols, screening efficiency is constrained by healthcare providers’ inadequate knowledge of screening protocols. Variations in the professional backgrounds of staff across departments lead to inconsistent understanding of DM-TB co-infection, differing criteria for screening indications, and non-uniform interpretation of results. These discrepancies ultimately result in missed screenings, misdiagnoses, and compromised screening outcomes (91). Therefore, clinical institutions must establish standardized bidirectional screening protocols that clearly define departmental responsibilities throughout the entire workflow, encompassing inpatient admission data collection, high-risk population identification, screening test selection, result feedback, positive case referral, and post-screening follow-up management. Furthermore, regular training and competency assessment programs are essential to enhance healthcare providers’ proficiency in screening procedures and improving overall screening efficiency (89–92).

## 7 Challenges and countermeasures in screening implementation

Implementing TB screening among diabetic populations faces multiple challenges. First, resources are unevenly distributed. Primary healthcare facilities frequently suffer from shortages of specialized personnel and diagnostic equipment, necessitating the development of simplified diagnostic tools suitable for grassroots healthcare settings and the enhancement of training for healthcare workers. The target locations for screening programs should be determined based on local epidemiological characteristics, geography, and demographics, with a focus on high-risk populations and key areas of concern. This process requires close collaboration with relevant communities, leaders, and policymakers to conduct assessments aligned with local public health priorities (93). Second, patient compliance issues. Patients often refuse TB screening due to dual disease stigma or insufficient disease awareness, particularly pronounced among low-income populations where financial constraints frequently result in low screening participation rates (94, 95). Additionally, cumbersome screening procedures or inconvenient access to healthcare services lead to low follow-up rates for patients with abnormal CXR findings, which impedes timely and accurate diagnosis. To mitigate these issues, health education efforts must be strengthened through community promotion and patient case management to improve health literacy. Selecting appropriate screening technologies, providing immediate interpretation of results, and reinforcing patients’ willingness to seek care proactively. Develop rigorous screening protocols to ensure screening coverage rates exceed 90%. For those with abnormal initial CXR findings, epidemiological investigation teams must deploy home visit personnel and arrange necessary transportation to facilitate sputum sample collection for diagnostic testing, thereby ensuring that confirmed TB patients receive timely medical care. Third, policy support remains a critical factor. Although the WHO recommends TB screening among diabetic patients, most countries lack integrated implementation mechanisms. Governments should establish unified, scientifically grounded, and consistently implemented policies, develop “diabetes-tailored TB screening algorithms” to customize screening protocols based on individual patient characteristics (96). Furthermore, electronic health record systems can be leveraged to facilitate interoperability between DM and TB clinical data, enabling healthcare providers to promptly identify high-risk individuals and enhance the accuracy of TB screening.

## 8 Follow-up and management after screening

### 8.1 Treatment management for confirmed tuberculosis patients

Follow-up and management are required after TB screening in diabetic patients. Confirmed cases should begin anti-tuberculosis treatment (DOTS regimen) and optimize glycemic control (target HbA1c < 7%) at the same time. Treatment adherence and adverse medication reactions (such as hepatotoxicity or nephrotoxicity) must be closely monitored during treatment. Diabetic patients have greater rates of treatment failure and relapse due to impaired immune function, requiring more frequent follow-up. For patients who interrupt treatment, sputum smear, culture, and drug susceptibility testing must be repeated to adjust the treatment regimen (97).

### 8.2 Preventive treatment for LTBI

According to WHO guidelines, 6 or 9 months of isoniazid (6H/9H), 3 months of isoniazid plus rifapentine (3HP), 3 months of isoniazid plus rifampin (3HR), or an alternative regimen including 1 month of isoniazid plus rifapentine (1HP) or 4 months of rifampin (4R) can be used to treat patients with LTBI (98). A prospective multicenter study by Huang et al. suggested that, from a public health perspective, older adult(s) diabetic patients with poorly controlled blood glucose should be prioritized for LTBI preventive treatment (99).

Drug interactions should be taken into account while treating individuals with DM-LTBI, and severe adverse drug responses should be prevented. Rifampicin, a potent inducer of hepatic microsomal enzymes (specifically the cytochrome P450 [CYP450] enzyme system), significantly reduces the plasma concentrations of sulfonylureas, glinides, thiazolidinediones, and other hypoglycemic agents. Dynamic blood glucose monitoring and adjustment of antidiabetic regimens are essential to ensure effective glycemic control (90). Among the WHO-recommended regimens, the 3HP regimen is widely accepted due to its shorter duration and simpler administration, leading to higher completion rates than the 9H regimen (100). However, the 3HP regimen raises safety concerns for diabetic individuals because rifapentine interferes with the metabolism of oral hypoglycemic drugs (101). Huang et al. also found that over three-quarters of diabetic patients in the 3HP group experienced adverse drug reactions, mostly mild (Grade 1 or 2), with a regimen completion rate of 84.1% (99). It should be noted that due to generally poor treatment adherence among diabetic patients, it remains unclear whether ultra-short-course LTBI preventive regimens can improve adherence, how drug interactions affect diabetes control, or what the protective efficacy is. More randomized controlled trials (RCTs) with high-quality evidence are required.

LTBI management can help improve medication adherence. AI-enabled smart pillboxes can automatically record dosing and send SMS reminders for missed doses, while also facilitating regular patient assessments for adverse events (102, 103). Follow-up management is still required after treatment discontinuation, typically for at least 2 years.

### 8.3 Establishing a health management model based on tuberculosis screening for diabetic patients

**Figure 1:**
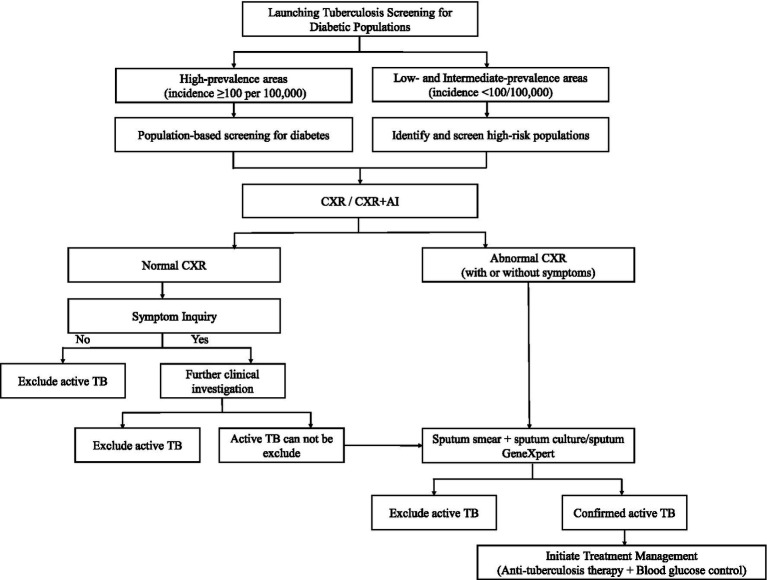
Flowchart for launching tuberculosis screening in diabetic populations. It distinguishes between high-prevalence and low-to intermediate-prevalence areas, recommending population-based or high-risk screenings. CXR or CXR+AI identifies normal or abnormal results. Normal CXR leads to symptom inquiry; “No” excludes TB, “Yes” may require further investigation. Abnormal CXR or symptoms lead to sputum tests. Outcomes include excluding or confirming active TB, with treatment involving anti-tuberculosis therapy and blood glucose control. For further assessment of LTBI infection status, LTBI screening may be conducted, though it is not strongly recommended. LTBI screening is only advised in high-prevalence areas.

Diabetes and tuberculosis influence each other, forming a vicious cycle. TB screening for diabetic patients should be routinely integrated into standard health management. To address this dual-disease burden, a comprehensive, synchronized, and intelligent DM-TB management system covering the entire care process from screening, diagnosis, treatment, to management is necessary to be established. This systematic, closed-loop approach can improve treatment outcomes and quality of life. In the screening stage, a TB risk assessment system for diabetic patients is recommended. By entering patient information, the system would automatically classify individuals into high-, medium-, or low-risk groups. Cost-effectiveness models could be incorporated to dynamically optimize screening resource allocation. During diagnosis, more accurate screening technologies should be developed. Using AI to assist in diagnosis can improve accuracy, shorten delays, and reduce missed or misdiagnosed cases. In the treatment phase, a coordinated care mechanism between endocrinology and infectious disease departments should be established to develop personalized treatment plans. This includes optimizing both anti-TB and glucose-lowering drugs while closely monitoring liver and kidney function, blood glucose levels, and adverse drug reactions to lower the risk of treatment failure. For long-term management, data exchange between diabetes electronic health records and TB prevention and control information registration systems should be promoted. This enables real-time dynamic updates of patient screening results, treatment progress, and follow-up records, forming a “data-driven, multidisciplinary collaboration” model for efficient management of diabetes with tuberculosis. The tuberculosis screening pathway for individuals with diabetes is shown in Figure 1.

## 9 Conclusion

Diabetes is a risk factor for tuberculosis in high TB burden settings, and individuals with poorly regulated glycaemia need particular attention. The vicious cycle of diabetes-tuberculosis co-morbidity has become an urgent public health challenge. Therefore, diabetic patients should be considered a key target group for TB screening, with differentiated screening strategies tailored to regional TB epidemiology. Future efforts should focus on developing and deploying novel screening tools that are simple, accurate, and low-cost to overcome current limitations in diagnostic efficiency. Patients with both diabetes and LTBI should receive preventive treatment where feasible, with improved coordinated management of both diabetes and tuberculosis.

## References

[1] Shu W Liu Y. Committed to innovation and striving for long-term progress: interpretation of research and innovation chapter in the global tuberculosis report 2024. Chin J Antituberc. (2025) 47:137–41. doi: 10.19982/j.issn.1000-6621.20240559

[2] Dooley KE Chaisson RE. Tuberculosis and diabetes mellitus: convergence of two epidemics. Lancet Infect Dis. (2009) 9:737–46. doi: 10.1016/S1473-3099(09)70282-8, PMID: 19926034 PMC2945809

[3] The End TB Strategy: a global rally. Lancet Respir Med.(2014) 2:943. doi: 10.1016/S2213-2600(14)70277-2 25466339

[4] Duncan BB Magliano DJ Boyko EJ. IDF diabetes atlas 11th edition 2025: global prevalence and projections for 2050. Nephrol Dial Transplant. (2025) 28:gfaf177. doi: 10.1093/ndt/gfaf177 40874767

[5] Al-Rifai RH Pearson F Critchley JA Abu-Raddad LJ. Association between diabetes mellitus and active tuberculosis: a systematic review and meta-analysis. PLoS One. (2017) 12:e0187967. doi: 10.1371/journal.pone.0187967, PMID: 29161276 PMC5697825

[6] Lee MR Huang YP Kuo YT Luo CH Shih YJ Shu CC . Diabetes mellitus and latent tuberculosis infection: a systematic review and metaanalysis. Clin Infect Dis. (2017) 64:719–27. doi: 10.1093/cid/ciw836, PMID: 27986673 PMC5399944

[7] Wu Q Liu Y Ma YB Liu K Chen SH. Incidence and prevalence of pulmonary tuberculosis among patients with type 2 diabetes mellitus: a systematic review and meta-analysis. Ann Med. (2022) 54:1657–66. doi: 10.1080/07853890.2022.2085318, PMID: 35703920 PMC9225779

[8] Awad SF Critchley JA Abu-Raddad LJ. Impact of diabetes mellitus on tuberculosis epidemiology in Indonesia: a mathematical modeling analysis. Tuberculosis (Edinb). (2022) 134:102164. doi: 10.1016/j.tube.2022.102164, PMID: 35288340

[9] Chen H Liu M Gu F. Meta-analysis on the co-morbidity rate between tuberculosis and diabetes mellitus in China. Chin J Epidemiol. (2013) 34:1128–33. doi: 10.3760/cma.j.issn.0254-6450.2013.011.019 24517949

[10] Kumar Nathella P Babu S. Influence of diabetes mellitus on immunity to human tuberculosis. Immunology. (2017) 152:13–24. doi: 10.1111/imm.12762, PMID: 28543817 PMC5543489

[11] Abbas U Masood KI Khan A Irfan M Saifullah N Jamil B. Tuberculosis and diabetes mellitus: relating immune impact of co-morbidity with challenges in disease management in high burden countries. J Clin Tuberc Other Mycobact Dis. (2022) 29:100343. doi: 10.1016/j.jctube.2022.100343, PMID: 36478777 PMC9720570

[12] Yang S Yan X. New advance in diabetes and tuberculosis: interrelation, diagnosis and treatment. Electron J Emerg Infect Dis. (2018) 3:234–8. doi: 10.3877/j.issn.2096-2738.2018.04.011

[13] World Health Organization. WHO consolidated guidelines on tuberculosis.Module 2:Screening-systematic screening for tuberculosis disease. Geneva: World Health Organization (2021). 33822560

[14] Zeng J Wang X Fang M Lu S. Interpretation for the third edition of WHO operational handbook on tuberculosis, module 6: tuberculosis and comorbidities. Clinical Focus. (2025) 40:270–4. doi: 10.3969/j.issn.1004-583X.2025.03.014

[15] Mave V Nimkar S Prasad H Kadam D Meshram S Lokhande R. Tuberculosis screening among persons with diabetes mellitus in Pune, India. BMC Infect Dis. (2017) 17:388. doi: 10.1186/s12879-017-2483-9, PMID: 28577535 PMC5457599

[16] Marks GB Nguyen NV Nguyen PTB Nguyen TA Nguyen HB Tran KH. Community-wide screening for tuberculosis in a high-prevalence setting. N Engl J Med. (2019) 381:1347–57. doi: 10.1056/NEJMoa1902129, PMID: 31577876

[17] Pealing L Wing K Mathur R Prieto-Merino D Smeeth L Moore DA. Risk of tuberculosis in patients with diabetes: population based cohort study using the UK clinical practice research datalink. BMC Med. (2015) 13:135. doi: 10.1186/s12916-015-0381-9, PMID: 26048371 PMC4470065

[18] Zhao W Shi L Fonseca VA He J Shao D Zhao J. Screening patients with type 2 diabetes for active tuberculosis in communities of China. Diabetes Care. (2013) 36:e159–60. doi: 10.2337/dc13-1007, PMID: 23970731 PMC3747870

[19] Wang J Xu X Cai X Yang M. Cost-effectiveness analysis of TB screening in patients with diabet. Chin J Antituberc. (2019) 41:968–73. doi: 10.3969/j.issn.1000-6621.2019.09.011

[20] Lin HH Ezzati M Murray M. Tobacco smoke, indoor air pollution and tuberculosis: a systematic review and meta-analysis. PLoS Med. (2007) 4:e20. doi: 10.1371/journal.pmed.0040020, PMID: 17227135 PMC1769410

[21] Dong B Ge N Liu G. Social economical status, behaviors and environment as the risk factors of tuberculosis in Chengdu China. Chin J Epidemiol. (2001) 22:102–4. doi: 10.3760/j.issn:0254-6450.2001.02.007 11860854

[22] Ji Y Cao H Liu Q Li Z Song H Xu D. Screening for pulmonary tuberculosis in high-risk groups of diabetic patients. Int J Infect Dis. (2020) 93:84–9. doi: 10.1016/j.ijid.2020.01.019, PMID: 31978585

[23] Lin YH Chen CP Chen PY Huang JC Ho C Weng HH . Screening for pulmonary tuberculosis in type 2 diabetes elderly: a cross-sectional study in a community hospital. BMC Public Health. (2015) 15:3. doi: 10.1186/1471-2458-15-3, PMID: 25572102 PMC4324855

[24] Chen Z Liu Q Song R Zhang W Wang T Lian Z. The association of glycemic level and prevalence of tuberculosis: a meta-analysis. BMC Endocr Disord. (2021) 21:123. doi: 10.1186/s12902-021-00779-6, PMID: 34134685 PMC8207612

[25] Franco JV Bongaerts B Metzendorf MI Risso A Guo Y Peña Silva L . Diabetes as a risk factor for tuberculosis disease. Cochrane Database Syst Rev. (2024) 8:CD016013. doi: 10.1002/14651858.CD016013.pub2, PMID: 39177079 PMC11342417

[26] Obels I Ninsiima S Critchley JA Huangfu P. Tuberculosis risk among people with diabetes mellitus in sub-Saharan Africa: a systematic review. Trop Med Int Health. (2022) 27:369–86. doi: 10.1111/tmi.13733, PMID: 35146851 PMC9303199

[27] Gao L Li X Liu J Wang X. Incidence of active tuberculosis in individuals with latent tuberculosis infection in rural China: follow-up results of a population-based, multicentre, prospective cohort study. Lancet Infect Dis. (2017) 17:1053–61. doi: 10.1016/S1473-3099(17)30402-4, PMID: 28716677

[28] Hewage S Somasundaram N Ratnasamy V Ranathunga I Fernando A Perera I. Active screening of patients with diabetes mellitus for pulmonary tuberculosis in a tertiary care hospital in Sri Lanka. PLoS One. (2021) 16:e0249787. doi: 10.1371/journal.pone.0249787, PMID: 33831095 PMC8031956

[29] Liu Q You N Wen J Wang J Ge Y Shen Y . Yield and efficiency of a population-based mass tuberculosis screening intervention among persons with diabetes in Jiangsu Province, China. Clin Infect Dis. (2023) 77:103–11. doi: 10.1093/cid/ciad118, PMID: 36869807 PMC10320136

[30] WHO. Consolidated guidelines on tuberculosis:Module 1:Prevention–tuberculosis preventive treatment, second edition. Geneva: World Health Organization (2024). 39298638

[31] Liu Q Yan W Liu R Bo E Liu J Liu M. The association between diabetes mellitus and the risk of latent tuberculosis infection: a systematic review and Meta-analysis. Front Med (Lausanne). (2022) 9:899821. doi: 10.3389/fmed.2022.899821, PMID: 35547228 PMC9082645

[32] Dabhi PA Thangakunam B Gupta R James P Thomas N Naik D. Screening for prevalence of current TB disease and latent TB infection in type 2 diabetes mellitus patients attending a diabetic clinic in an Indian tertiary care hospital. PLoS One. (2020) 15:e0233385. doi: 10.1371/journal.pone.0233385, PMID: 32502176 PMC7274437

[33] Torres AV Corrêa RDS Bevilacqua MF do Prado LCF Bandeira FMGC Rodrigues LS . Screening of latent tuberculosis infection among patients with diabetes mellitus from a high-burden area in Brazil. Front Clin Diabetes Healthc. (2022) 3:914574. doi: 10.3389/fcdhc.2022.914574 36992754 PMC10012069

[34] Jeong YJ Lee KS. Pulmonary tuberculosis: up-to-date imaging and management. AJR Am J Roentgenol. (2008) 191:834–44. doi: 10.2214/AJR.07.3896, PMID: 18716117

[35] Pires DC Arueira Chaves L Dantas Cardoso CH Faria LV Rodrigues Campos S Sobreira da Silva MJ. Effects of low dose computed tomography (LDCT) on lung cancer screening on incidence and mortality in regions with high tuberculosis prevalence: a systematic review. PLoS One. (2024) 19:e0308106. doi: 10.1371/journal.pone.0308106, PMID: 39259749 PMC11389911

[36] Habib SS Rafiq S Zaidi SMA Ferrand RA Creswell J Van Ginneken B . Evaluation of computer aided detection of tuberculosis on chest radiography among people with diabetes in Karachi Pakistan. Sci Rep. (2020) 10:6276. doi: 10.1038/s41598-020-63084-7, PMID: 32286389 PMC7156514

[37] Emoru RD Mrema LE Ntinginya NE Biraro IA van Crevel R Critchley JA. Accuracy of computer-aided chest x-ray interpretation for tuberculosis screening in people with diabetes mellitus: a systematic review. Trop Med Int Health. (2025) 30:323–31. doi: 10.1111/tmi.14103, PMID: 40084399

[38] Liu M Liu Y Li L. Research and application progress of artificial intelligence-assisted imaging diagnosis in tuberculosis prevention and control. Prev Med Trib. (2024) 30:230–235, 240. doi: 10.16406/j.pmt.issn.1672-9153.2024.3.015

[39] Chen Z Zhou G. Application of GeneXpert MTB/RIF technology in the diagnosis of tuberculosis and detection of rifampicin resistance. Int J Epidemiol Infect Dis. (2017) 44:420–4. doi: 10.3760/cma.j.issn.1673-4149.2017.06.015

[40] Witarto AB Ceachi B Popp C Zurac S Daha IC Sari FE. AI-based analysis of Ziehl-Neelsen-stained sputum smears for *Mycobacterium tuberculosis* as a screening method for active tuberculosis. Life (Basel). (2024) 14:1418. doi: 10.3390/life14111418, PMID: 39598216 PMC11595674

[41] Chegou NN Hoek KG Kriel M Warren RM Victor TC Walzl G. Tuberculosis assays: past, present and future. Expert Rev Anti-Infect Ther. (2011) 9:457–69. doi: 10.1586/eri.11.23, PMID: 21504402

[42] World Health Organization. WHO consolidated guidelines on tuberculosis: Module 3: Diagnosis – Rapid diagnostics for tuberculosis detection. Geneva: World Health Organization (2020). 33999549

[43] Byanyima P Kaswabuli S Musisi E Nabakiibi C Zawedde J Sanyu I. Feasibility and sensitivity of saliva GeneXpert MTB/RIF ultra for tuberculosis diagnosis in adults in Uganda. Microbiol Spectr. (2022) 10:e0086022. doi: 10.1128/spectrum.00860-22, PMID: 36154664 PMC9603304

[44] Deshmukh S Atre S Chavan A Raskar S Sawant T Mave V. Assessment of the Xpert assay among adult pulmonary tuberculosis suspects with and without diabetes mellitus. Int J Tuberc Lung Dis. (2020) 24:113–7. doi: 10.5588/ijtld.19.0239, PMID: 32005314

[45] Shete PB Farr K Strnad L Gray CM Cattamanchi A. Diagnostic accuracy of TB-LAMP for pulmonary tuberculosis: a systematic review and meta-analysis. BMC Infect Dis. (2019) 19:268. doi: 10.1186/s12879-019-3881-y, PMID: 30890135 PMC6425614

[46] Tayal D Sethi P Jain P. Point-of-care test for tuberculosis: a boon in diagnosis. Monaldi Arch Chest Dis. (2023) 94. doi: 10.4081/monaldi.2023.2528 37114932

[47] Church EC Steingart KR Cangelosi GA Ruhwald M Kohli M Shapiro AE. Oral swabs with a rapid molecular diagnostic test for pulmonary tuberculosis in adults and children: a systematic review. Lancet Glob Health. (2024) 12:e45–54. doi: 10.1016/S2214-109X(23)00469-2, PMID: 38097297 PMC10733129

[48] Andama A Whitman GR Crowder R Reza TF Jaganath D Mulondo J. Accuracy of tongue swab testing using Xpert MTB-RIF ultra for tuberculosis diagnosis. J Clin Microbiol. (2022) 60:e0042122. doi: 10.1128/jcm.00421-22, PMID: 35758702 PMC9297831

[49] Mou L Wiwatpanit T Piriyapol A Chawengkul P Thaipadungpanit J Kulchaitanaroaj P. Early health technology assessment of tongue swab for non-sputum based pulmonary tuberculosis diagnosis in Thailand. Lancet Reg Health Southeast Asia. (2025) 33:100533. doi: 10.1016/j.lansea.2025.100533, PMID: 39945002 PMC11814696

[50] Pang Y Li L. Tongue swabs: a novel tool for tuberculosis screening. Zhonghua Jie He He Hu Xi Za Zhi. (2025) 48:605–8. Chinese. doi: 10.3760/cma.j.cn112147-20250214-00085 40582972

[51] Ealand CS Sewcharran A Peters JS Gordhan BG Kamariza M Bertozzi CR. The performance of tongue swabs for detection of pulmonary tuberculosis. Front Cell Infect Microbiol. (2023) 13:1186191. doi: 10.3389/fcimb.2023.1186191, PMID: 37743867 PMC10512057

[52] Luabeya AK Wood RC Shenje J Filander E Ontong C Mabwe S. Noninvasive detection of tuberculosis by Oral swab analysis. J Clin Microbiol. (2019) 57:e01847–18. doi: 10.1128/JCM.01847-18, PMID: 30541931 PMC6425180

[53] Xia H Zhao Y. The potentials and challenges of using tongue swab for *Mycobacterium tuberculosis* complex detection through nucleic acid amplification tests in the diagnosis and screening of pulmonary tuberculosis. Chin J Antituberc. (2025) 47:976–80. doi: 10.19982/j.issn.1000-6621.20250124

[54] Tuberculosis Control Branch of Chinese Antituberculosis Association, Standardization Professional Branch of Chinese Antituberculosis Association, Elderly Tuberculosis Control Branch of Chinese Antituberculosis Association Zhang H Gao L Cheng J. Expert consensus on the application of *Mycobacterium tuberculosis* infection detection technologies. Chin J Antituberc. (2025) 47:813–29. doi: 10.19983/j.issn.2096-8493.20252001

[55] Song Q Guo H Zhong H Liu Z Chen X Wang C. Evaluation of a new interferon-gamma release assay and comparison to tuberculin skin test during a tuberculosis outbreak. Int J Infect Dis. (2012) 16:e522–6. doi: 10.1016/j.ijid.2012.03.003, PMID: 22542006

[56] Liu CW Ma LD Wei ZH Jiang SP Li B Wang Y . Application and evaluation of γ-interferon releasing test in tuberculosis screening in general practice outpatients. Electron J Emerg Infect Dis. (2023) 8:63–8. doi: 10.19871/j.cnki.xfcrbzz.2023.06.012

[57] Gupta A Chandra E Anand S Kumar N Arora R Rana D . Latent tuberculosis diagnostics: current scenario and review. Monaldi Arch Chest Dis. (2025) 95. doi: 10.4081/monaldi.2024.2984, PMID: 38700134

[58] Yang Y Wang HJ Hu WL Bai GN Hua CZ. Diagnostic value of interferon-gamma release assays for tuberculosis in the immunocompromised population. Diagnostics (Basel). (2022) 12:453. doi: 10.3390/diagnostics12020453, PMID: 35204544 PMC8871457

[59] Dong X Guo H Zhang C Wang J He J Li G . Application value of two-step detection of *Mycobacterium tuberculosis* infection screening in schools. Chin J Antituberc. (2022) 44:802–7. doi: 10.19982/j.issn.1000-6621.20220096

[60] Erkens CG Dinmohamed AG Kamphorst M Toumanian S van Nispen-Dobrescu R Alink M . Added value of interferon-gamma release assays in screening for tuberculous infection in the Netherlands. Int J Tuberc Lung Dis. (2014) 18:413–20. doi: 10.5588/ijtld.13.0589, PMID: 24670695

[61] Internal Clinical Guidelines Team (UK). Tuberculosis: Prevention, Diagnosis, Management and service organisation. London: National Institute for Health and Care Excellence (UK) (2016). 26820019

[62] Schaberg T Bauer T Brinkmann F Diel R Feiterna-Sperling C Haas W . S2k-Leitlinie: Tuberkulose im Erwachsenenalter [tuberculosis guideline for adults - guideline for diagnosis and treatment of tuberculosis including LTBI testing and treatment of the German central committee (DZK) and the German respiratory society (DGP)]. Pneumologie. (2017) 71:325–97. German. doi: 10.1055/s-0043-105954 28651293

[63] Hamada Y Gupta RK Quartagno M Izzard A Acuna-Villaorduna C Altet N. Predictive performance of interferon-gamma release assays and the tuberculin skin test for incident tuberculosis: an individual participant data meta-analysis. EClinicalMedicine. (2023) 56:101815. doi: 10.1016/j.eclinm.2022.101815, PMID: 36636295 PMC9829704

[64] Chinese Antituberculosis Association, Schools and Children Branch of the Chinese Antituberculosis Association, Editorial Board of Chinese Journal of Antituberculosis. Expert consensus of clinical application of the recombinant *Mycobacterium tuberculosis* fusion protein (EC). Chin J Antituberc. (2020) 42:761–8. doi: 10.3969/i.issn.1000-6621.2020.08.001

[65] World Health Organization. Rapid communication: TB antigen-based skin tests for the diagnosis of TB infection (WHO-UCN-TB-2022.1). (2022) Available online at: https://apps.who.int/iris/handle/10665/352802

[66] Chen J Zhao L Zhou X Mo Y Kong L Ma F. Safety and diagnostic performance of recombinant fusion protein ESAT6-CPF10 skin test in a large population: a phase 4 clinical trial. J Infect Dis. (2025) 232:882–91. doi: 10.1093/infdis/jiaf332, PMID: 40560769

[67] Diao S Liu Z Liu D Cheng X Zeng L Jiao XF. Long-term economic evaluation of the recombinant *Mycobacterium tuberculosis* fusion protein (EC) test for the diagnosis of *Mycobacterium tuberculosis* infection. Front Pharmacol. (2023) 14:1161526. doi: 10.3389/fphar.2023.1161526, PMID: 37261290 PMC10228647

[68] Zhao A Kang W Wang G Gao Z Du W Lu J . Screening for latent tuberculosis infection and identification of BCG vaccination by recombinant *Mycobacterium tuberculosis* 11 kDa. Chin J Antituberc. (2020) 42:821–5. doi: 10.3969/j.issn.1000-6621.2020.08.008

[69] Su Q Wang Q Zhang T Liu Y. Comparison of recombinant *Mycobacterium tuberculosis* fusion protein skin test and tuberculin skin test for screening latent tuberculosis infection in school students. Chin J Infect Control. (2023) 22:547–51. doi: 10.12138/j.issn.1671-9638.20233724

[70] Bulterys MA Wagner B Redard-Jacot M Suresh A Pollock NR Moreau E . Point-of-care urine LAM tests for tuberculosis diagnosis: a status update. J Clin Med. (2019) 9:111. doi: 10.3390/jcm9010111, PMID: 31906163 PMC7020089

[71] Zhang TH Ma ZC Liu RM Shang YY Ma LP Han M . Evaluation of the efficacy of urine-based lipoarabinomannan antigen test in the diagnosis of pulmonary tuberculosis. Zhonghua Jie He He Hu Xi Za Zhi. (2024) 47:132–6. Chinese. doi: 10.3760/cma.j.cn112147-20230814-00074 38309962

[72] Kerkhoff AD Sossen B Schutz C Reipold EI Trollip A Moreau E. Diagnostic sensitivity of SILVAMP TB-LAM (FujiLAM) point-of-care urine assay for extra-pulmonary tuberculosis in people living with HIV. Eur Respir J. (2020) 55:1901259. doi: 10.1183/13993003.01259-2019, PMID: 31699835 PMC7002975

[73] Huang L Niu Y Zhang L Yang R Wu M. Diagnostic value of chemiluminescence for urinary lipoarabinomannan antigen assay in active tuberculosis: insights from a retrospective study. Front Cell Infect Microbiol. (2023) 13:1291974. doi: 10.3389/fcimb.2023.1291974, PMID: 38145052 PMC10748405

[74] Kerschberger B Ntshalintshali N Mpala Q Díaz Uribe PA Maphalala G Kalombola S. Field suitability and diagnostic accuracy of the biocentric open real-time PCR platform for dried blood Spot-based HIV viral load quantification in Eswatini. J Acquir Immune Defic Syndr. (2019) 82:96–104. doi: 10.1097/QAI.0000000000002101, PMID: 31408452 PMC6727953

[75] Ekeke N Aniwada E Chukwu J Nwafor C Meka A Chukwuka A . Screening diabetes mellitus patients for tuberculosis in southern Nigeria: a pilot study. Adv Respir Med. (2020) 88:6–12. doi: 10.5603/ARM.2020.0072, PMID: 32153002

[76] Basir MS Habib SS Zaidi SMA Khowaja S Hussain H Ferrand RA. Operationalization of bi-directional screening for tuberculosis and diabetes in private sector healthcare clinics in Karachi, Pakistan. BMC Health Serv Res. (2019) 19:147. doi: 10.1186/s12913-019-3975-7, PMID: 30841929 PMC6404337

[77] India Diabetes Mellitus--Tuberculosis Study Group. Screening of patients with diabetes mellitus for tuberculosis in India. Trop Med Int Health. (2013) 18:646–54. doi: 10.1111/tmi.12083 23448175

[78] Alisjahbana B McAllister SM Ugarte-Gil C Panduru NM Ronacher K Koesoemadinata RC . Screening diabetes mellitus patients for pulmonary tuberculosis: a multisite study in Indonesia, Peru, Romania and South Africa. Trans R Soc Trop Med Hyg. (2021) 115:634–43. doi: 10.1093/trstmh/traa100, PMID: 33118039

[79] Majumder A Carroll B Bhana S Tefu D Syeda S Martinson N. Screening for active tuberculosis in a diabetes mellitus clinic in Soweto, South Africa. Int J Tuberc Lung Dis. (2016) 20:992–3. doi: 10.5588/ijtld.16.0340, PMID: 27287661

[80] Koesoemadinata RC Kranzer K Livia R Susilawati N Annisa J Soetedjo NNM. Computer-assisted chest radiography reading for tuberculosis screening in people living with diabetes mellitus. Int J Tuberc Lung Dis. (2018) 22:1088–94. doi: 10.5588/ijtld.17.0827, PMID: 30092877

[81] Eneogu RA Mitchell EMH Ogbudebe C Aboki D Anyebe V Dimkpa CB. Iterative evaluation of mobile computer-assisted digital chest x-ray screening for TB improves efficiency, yield, and outcomes in Nigeria. PLOS Glob Public Health. (2024) 4:e0002018. doi: 10.1371/journal.pgph.0002018, PMID: 38232129 PMC10793917

[82] Emery JC Dodd PJ Banu S Frascella B Garden FL Horton KC. Estimating the contribution of subclinical tuberculosis disease to transmission: An individual patient data analysis from prevalence surveys. eLife. (2023) 12:e82469. doi: 10.7554/eLife.82469, PMID: 38109277 PMC10727500

[83] Prakoso DA Istiono W Mahendradhata Y Arini M. Acceptability and feasibility of tuberculosis-diabetes mellitus screening implementation in private primary care clinics in Yogyakarta, Indonesia: a qualitative study. BMC Public Health. (2023) 23:1908. doi: 10.1186/s12889-023-16840-z, PMID: 37789310 PMC10546762

[84] Abreu RG Sousa AIA Oliveira MRF Sanchez MN. Tuberculosis and diabetes: probabilistic linkage of databases to study the association between both diseases. Epidemiol Serv Saude. (2017) 26:359–68 English, Portuguese. doi: 10.5123/S1679-49742017000200013, PMID: 28492777

[85] MacPherson P Shanaube K Phiri MD Rickman HM Horton KC Feasey HRA. Community-based active-case finding for tuberculosis: navigating a complex minefield. BMC Glob Public Health. (2024) 2:9. doi: 10.1186/s44263-024-00042-9, PMID: 39681899 PMC11622870

[86] Trinidad RM Brostrom R Morello MI Montgomery D Thein CC Gajitos ML. Tuberculosis screening at a diabetes clinic in the Republic of the Marshall Islands. J Clin Tuberc Other Mycobact Dis. (2016) 5:4–7. doi: 10.1016/j.jctube.2016.10.001, PMID: 31723690 PMC6850250

[87] van Crevel R Critchley JA. The interaction of diabetes and tuberculosis: translating research to policy and practice. Trop Med Infect Dis. (2021) 6:8. doi: 10.3390/tropicalmed6010008, PMID: 33435609 PMC7838867

[88] Saeedi P Petersohn I Salpea P Malanda B Karuranga S Unwin N . Global and regional diabetes prevalence estimates for 2019 and projections for 2030 and 2045: results from the international diabetes federation diabetes atlas, 9th edition. Diabetes Res Clin Pract. (2019) 157:107843. doi: 10.1016/j.diabres.2019.107843, PMID: 31518657

[89] Nyirenda JLZ Wagner D Ngwira B Lange B. Bidirectional screening and treatment outcomes of diabetes mellitus (DM) and tuberculosis (TB) patients in hospitals with measures to integrate care of DM and TB and those without integration measures in Malawi. BMC Infect Dis. (2022) 22:28. doi: 10.1186/s12879-021-07017-3, PMID: 34983434 PMC8725264

[90] National Clinical Research Center for Infectious Disease, The Third People’s Hospital of Shenzhen, National Clinical Research Center for Metabolic Disease, The Second Xiangya Hospital of Central South University, Chinese Antituberculosis Association, Editorial Board of Chinese Journal of Antituberculosis. Expert consensus on treatment and management of tuberculosis-diabetes mellitus (2021) 43:12–22. doi: 10.3969/j.issn.1000-6621.2021.01.004,

[91] Salifu RS Hlongwana KW. Barriers and facilitators to bidirectional screening of TB-DM in Ghana: healthcare workers' perspectives. PLoS One. (2020) 15:e0235914. doi: 10.1371/journal.pone.0235914, PMID: 32663233 PMC7360027

[92] Riza AL Pearson F Ugarte-Gil C Alisjahbana B van de Vijver S Panduru NM. Clinical management of concurrent diabetes and tuberculosis and the implications for patient services. Lancet Diabetes Endocrinol. (2014) 2:740–53. doi: 10.1016/S2213-8587(14)70110-X, PMID: 25194887 PMC4852378

[93] Taylor M Medley N van Wyk SS Oliver S. Community views on active case finding for tuberculosis in low- and middle-income countries: a qualitative evidence synthesis. Cochrane Database Syst Rev. (2024) 3:CD014756. doi: 10.1002/14651858.CD014756.pub2, PMID: 38511668 PMC10955804

[94] Biermann O Lönnroth K Caws M Viney K. Factors influencing active tuberculosis case-finding policy development and implementation: a scoping review. BMJ Open. (2019) 9:e031284. doi: 10.1136/bmjopen-2019-031284, PMID: 31831535 PMC6924749

[95] Buregyeya E Atusingwize E Sekandi JN Mugambe R Nuwematsiko R Atuyambe L. Developing strategies to address barriers for tuberculosis case finding and retention in care among refugees in slums in Kampala, Uganda: a qualitative study using the COM-B model. BMC Infect Dis. (2022) 22:301. doi: 10.1186/s12879-022-07283-9, PMID: 35346094 PMC8962141

[96] Ayakaka I Ackerman S Ggita JM Kajubi P Dowdy D Haberer JE. Identifying barriers to and facilitators of tuberculosis contact investigation in Kampala, Uganda: a behavioral approach. Implement Sci. (2017) 12:33. doi: 10.1186/s13012-017-0561-4, PMID: 28274245 PMC5343292

[97] Rabahi MF Silva Júnior JLRD Ferreira ACG Tannus-Silva DGS Conde MB. Tuberculosis treatment. J Bras Pneumol. (2017) 43:472–86. doi: 10.1590/S1806-37562016000000388, PMID: 29340497 PMC5792048

[98] WHO. Latent tuberculosis infection:Updated and consolidated guidelines for programmatic management. Geneva: World Health Organization (2018). 30277688

[99] Huang HL Huang WC Lin KD Liu SS Lee MR Cheng MH. Completion rate and safety of programmatic screening and treatment for latent tuberculosis infection in elderly patients with poorly controlled diabetic mellitus: a prospective multicenter study. Clin Infect Dis. (2021) 73:e1252–60. doi: 10.1093/cid/ciab209, PMID: 33677558 PMC8442788

[100] Njie GJ Morris SB Woodruff RY Moro RN Vernon AA Borisov AS. Isoniazid-Rifapentine for latent tuberculosis infection: a systematic review and Meta-analysis. Am J Prev Med. (2018) 55:244–52. doi: 10.1016/j.amepre.2018.04.030, PMID: 29910114 PMC6097523

[101] Zheng C Hu X Zhao L Hu M Gao F. Clinical and pharmacological hallmarks of rifapentine's use in diabetes patients with active and latent tuberculosis: do we know enough? Drug Des Devel Ther. (2017) 11:2957–68. doi: 10.2147/DDDT.S146506, PMID: 29066867 PMC5644564

[102] Yoopetch P Anothaisintawee T Gunasekara ADM Jittikoon J Udomsinprasert W Thavorncharoensap M. Efficacy of anti-tuberculosis drugs for the treatment of latent tuberculosis infection: a systematic review and network meta-analysis. Sci Rep. (2023) 13:16240. doi: 10.1038/s41598-023-43310-8, PMID: 37758777 PMC10533889

[103] Padmapriyadarsini C Sachdeva KS Nair D Ramachandran R. The paradigm shift in the approach to management of latent tuberculosis infection in high tuberculosis burden countries. Expert Rev Respir Med. (2021) 15:899–910. doi: 10.1080/17476348.2021.1862652, PMID: 33302729

